# Association of choroidal blood flow with autonomic dysfunction in patients with normal tension glaucoma

**DOI:** 10.1038/s41598-022-09162-4

**Published:** 2022-03-24

**Authors:** Da Young Shin, Kyung Euy Hong, Na Young Lee, Chan Kee Park, Hae Young L. Park

**Affiliations:** 1 Department of Ophthalmology, Eunpyeong St. Mary’s Hospital, College of Medicine, The Catholic University of Korea, Seoul, Republic of Korea; 2 Department of Ophthalmology, Seoul St. Mary’s Hospital, College of Medicine, the Catholic University of Korea, 222 Banpo-daero, Seocho-gu, Seoul, 06591 Republic of Korea

**Keywords:** Diseases, Pathogenesis, Risk factors

## Abstract

There is increasing evidence that autonomic dysfunction is an important factor in the progression of glaucoma. Mechanism of the association between autonomic dysfunction and progression of glaucoma is poorly understood. Since blood circulation is basically regulated by the autonomic nervous system, autonomic dysfunction may contribute to unstable or fluctuating blood pressure. Therefore, It is hypothesized that autonomic dysfunction may contribute to impaired ocular blood flow and lead to glaucoma progression. However, no clinical study yet has evaluated the relationship between ocular blood flow and autonomic nervous function. We enrolled 152 open angle glaucoma patient. Ocular blood flow was assessed by measuring vessel density (VD) using optical coherence tomography angiography, and autonomic nervous function was evaluated with heart-rate variability (HRV) parameters. The low frequency/high frequency (LF/HF) ratio, which is one of the HRV parameters, quantified the degree of sympathovagal balance. This indicator could represent autonomic dysfunction. Higher LF/HF ratio was associated with reduction of the deep parapapillary VD (R =  − 0.243, P = 0.003). Linear regression analysis showed a significant negative association between parapapillary choroidal VD and LF/HF ratio (β =  − 0.249; 95% confidential interval =  − 1.193 to − 0.249; P = 0.002) in multivariate analysis. We demonstarted the association between impaired ocular blood flow (parapapillary choroidal vessel density) and autonomic dysfunction (LF/HF ratio). This study could help understand the role of the autonomic dysfunction in pathophysiology of glaucoma progression.

## Introduction

Although elevated intraocular pressure (IOP) is considered a major risk factor for the development and progression of glaucoma, vascular dysregulation and perfusion abnormalities have also been considered as important factors in the progression of glaucoma^1,2^. The potential roles of the blood flow in the pathophysiology of glaucoma have been extensively investigated. Previous studies have reported that ocular blood flow was reduced in the optic nerve head (ONH), retina, choroid, and retrobulbar region in glaucoma^3–5^. Of systemic conditions, vascular diseases, such as low arterial blood pressure (BP) with nocturnal hypotension, migraines, Raynaud’s syndrome, and obstructive sleep apnea have been reported to be associated with glaucoma^6–8^.

Dysfunction of the autonomic nervous system (ANS) is one possible cause leading to insufficient and unstable blood flow. Blood flow abnormalities in vessels of the ONH lead to exacerbation of glaucoma. Many reports have investigate the association between autonomic dysfunction and glaucoma. Several studies have reported that abnormal autonomic nervous system (ANS) function was associated with increased risk for progression of glaucoma. Furthermore, autonomic neuropathies (sympathetic and parasympathetic) have been reported in patients with primary open-angle glaucoma and normal-tension glaucoma (NTG)^9–11^.

Measurement of heart rate variability (HRV) is a well-known tool, that is widely used to evaluate the ANS^12,13^. HRV permits study of the autonomic modulation of the heart’s sympathovagal balance. In previous studies, HRV has been used to evaluate autonomic dysfunction in glaucoma patients^14–18^. These studies reported that abnormal HRV parameters were associated with high prevalence of NTG and faster rates of central visual field (VF) loss in glaucoma^14,18–20^.

With the advent of optical coherence tomography angiography (OCT-A), we can visualize the vessel density (VD) in various retinal layers, which could be a surrogate for measuring blood flow^21^. In particular, lower parapapillary choroidal VD has been reported to be associated with the progression of glaucoma^22–24^. Investigations are ongoing to discover systemic risk factors that may influence impairment or loss of VD in glaucoma^25,26^. Dips in nocturnal BP have been reported to be associated with localized choroidal VD loss in glaucoma patients^27^. Furthermore, glaucoma patients with higher systemic BP showed reducted macular VD^28^. These studies could help us determine the relationship between systemic conditions and blood flow to the ONH or to the retina and help us understand the pathophysiology of progression in glaucoma.

In this study, we investigate the association between autonomic dysfunction and impaired ocular blood flow in glaucoma. Ocular blood flow was assessed by measuring VD using OCT-A, and autonomic dysfunction was evaluated with HRV parameters. We aimed to determine whether HRV parameters and autonomic imbalance are associated with VD status in patients with glaucoma.

## Results

A total of 152 glaucoma patients (152 eyes) were included in this study. The demographics features of the subjects are presented in Table 1. We measured the correlations between the mean deviation (MD) or pattern standard deviation (PSD) of VFs, and the HRV parameters. Worse MDs or PSDs of the VFs were correlated with larger IOP fluctuations (R = −0.208, P = 0.011, and R = 0.162, P = 0.049, respectively, Table 2). Lower MD values were correlated with thinner superficial VD in the macular (R = 0.209, P = 0.015) and parapapillary (R = 0.421, P = 0.001) regions (Table 2).

**Table 1:** Demographics of patients with glaucoma.

Variables	152 eyes
Age, y	50.97 $\pm$ 11.15
Sex, male/female	58/94
Hypertension, n (%)	21(13.8%)
Diabetics, n (%)	2(1.3%)
Migraine, n (%)	22(14.5%)
History of refractive surgery, n (%)	11(7.2%)
CCT, $\mathrm{\mu m}$	50.97 $\pm$ 11.15
Disc hemorrhage, n (%)	22(14.5%)
Axial length, mm	25.91 $\pm$ 1.73
Mean IOP, mmHg	13.91 $\pm$ 2.61
IOP fluctuation, mmHg	4.71 $\pm$ 2.31
**Visual field**	
Mean deviation, dB	−3.14 $\pm$ 5.00
Pattern standard deivation, dB	5.48 $\pm$ 4.02
**Heart rate variability**	
SDNN	33.77 $\pm$ 18.41
LH Norm	50.09 $\pm$ 23.46
HF Norm	48.70 $\pm$ 24.81
LF/HF ratio	1.67 $\pm$ 1.63
**Angio OCT vessel density**	
Macular VD(superficial)	37.44 $\pm$ 3.02
Macular VD(deep)	44.11 $\pm$ 2.44
Parapapillary VD(superficial)	39.41 $\pm$ 4.60
Parapapillary VD(deep)	57.15 $\pm$ 5.02

CCT = central corneal thickness; IOP = intraocular pressure; SDNN = Standard deviation of NN-interval; LF = Low frequency ; HF = High frequency ; VD = Vessel density; OCT = Optical coherence tomography.

Continuous data are mean $\pm$ mean standard deviation unless otherwise indicated.

**Table 2:** Correlation coefficients for mean deviation, pattern standard deivation of visual field with ocular parameters and heart rate variability in total subjects.

	Mean deivation of visual field		Pattern standard deivation of visual field	
	R	P value*	R	P value*
Hypertension	−0.117	0.156	−0.046	0.121
Diabetics	−0.055	0.505	0.095	0.249
Migraine	0.033	0.375	−0.026	0.770
Mean IOP	0.104	0.206	−0.144	0.079
IOP fluctuation	−**0.208**	**0.011**	**0.162**	**0.049**
Disc hemorrhage	0.051	0.541	0.149	0.024
Axial length	−0.079	0.285	−0.019	0.832
CCT	0.038	0.667	−0.008	0.932
**Heart rate variability**				
SDNN	0.112	0.176	−0.123	0.135
LF Norm	−0.026	0.752	0.009	0.915
HF Norm	0.094	0.254	−0.138	0.940
LF/HF ratio	−0.034	0.679	0.037	0.651
**Angio OCT vessel density**				
Macular VD(superficial)	**0.209**	**0.015**	−0.162	0.061
Macular VD(deep)	0.038	0.664	−0.086	0.324
Parapapillary VD(superficial)	**0.421**	**0.001**	−**0.250**	**0.003**
Parapapillary VD(deep)	−0.160	0.053	0.160	0.053

IOP = intraocular pressure; SDNN = Standard deviation of NN-interval; LF = Low frequency ; HF = High frequency ; OCT = Optical coherence tomography; VD = Vessel density.

R = correlation coeffcient.

*Pearson correlation analysis.

We also measured the correlations between VD and the ocular parameters (Tables 3, 4). Increased age was correlated with superficial VD reduction in the macular (R = −0.171, P = 0.044) and parapapillary (R = −0.217, P = 0.009) regions. Reduced superficial parapapillary VD was associated with longer axial length (R = −0.326, P < 0.001) and greater IOP fluctuations (R = −0.191, P = 0.021). The reduction of deep parapapillary VD was associated with higher low frequency (LF) / high frequency (HF) ratio (R = −0.243, P = 0.003). Deep macular VD did not show significant correlations with the other parameters.

**Table 3:** Correlation coefficients for macular vessel density with ocular parameters and heart rate variability in total subjects.

	Macular VD (superficial)		Macular VD (deep)	
	R	P value*	R	P value*
Age	−**0.171**	**0.044**	−0.112	0.192
Sex	0.167	0.050	0.062	0.473
Axial length, mm	0.005	0.954	0.008	0.934
CCT	−0.020	0.817	−0.055	0.549
Hypertension	0.001	0.992	−0.147	0.084
Diabetics	−0.044	0.610	−0.023	0.793
Migraine	−0.050	0.559	−0.123	0.151
Disc hemorrhage	0.050	0.559	−0.069	0.420
Mean IOP	0.178	0.198	0.006	0.966
IOP fluctuation	−0.011	0.894	−0.041	0.633
**Heart rate variability**				
SDNN	−0.070	0.413	−0.045	0.602
LF Norm	−0.070	0.416	0.050	0.560
HF Norm	0.026	0.764	−0.071	0.410
LF/HF ratio	−0.141	0.099	−0.019	0.829

VD = Vessel density; IOP = intraocular pressure; SDNN = Standard deviation of NN-interval; LF = Low frequency ; HF = High frequency.

R = correlation coeffcient.

*Pearson correlation analysis.

**Table 4:** Correlation coefficients for parapapillary vessel density(VD) with ocular parameters and heart rate variability in total subjects.

	Parapapillary VD (superficial)		Parapapillary VD (deep)	
	R	P value*	R	P value*
Age	−**0.217**	**0.009**	0.007	0.934
Sex	0.110	0.188	−0.014	0.863
Axial length	−**0.326**	** < 0.001**	0.109	0.214
CCT	0.036	0.693	0.090	0.306
Hypertension	−0.120	0.152	−0.006	0.945
Diabetics	−0.084	0.314	0.001	0.992
Migraine	0.216	0.009	−0.020	0.808
Disc hemorrhage	0.156	0.061	−0.082	0.318
Mean IOP	−0.014	0.914	−0.172	0.178
IOP fluctuation	−**0.191**	**0.021**	−0.069	0.398
**Heart rate variability**				
SDNN	0.030	0.716	−0.037	0.651
LF Norm	−0.153	0.066	0.041	0.619
HF Norm	0.119	0.155	0.135	0.100
LF/HF ratio	−0.109	0.193	−**0.243**	**0.003**

VD = Vessel density; IOP = intraocular pressure; SDNN = Standard deviation of NN-interval; LF = Low frequency ; HF = High frequency ;

R = correlation coeffcient.

*Pearson correlation analysis.

**Figure 1:**
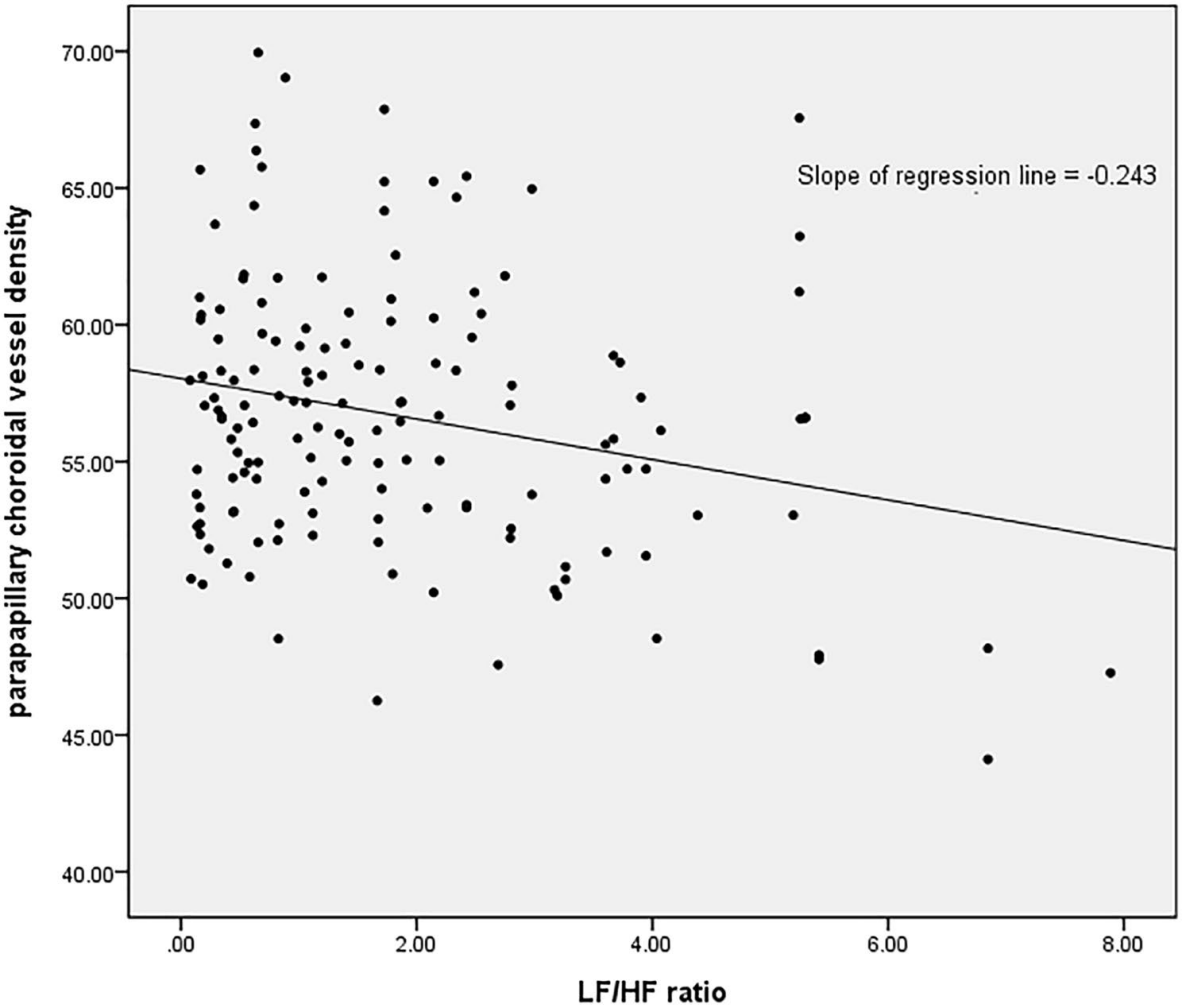
Scatter plot showing the relationships between the parapapillary choroidal vessel density and LF/HF ratio.

To determine factors related to parapapillary choroidal VD, regression analyses were performed. Lower parapapillary choroidal VD was associated with higher LF/HF ratio (β = −0.249, 95% confidential interval = −1.193 to −0.249; P = 0.002) in multivariate analysis (Table 5). Figure 1 shows a scatter plot between parapapillary choroidal VD and LF/HF ratio, which had a negative relationship.

**Table 5:** Regression analysis of factors associated with the parapapillary choroid vessel density.

Parapapillary choroid vessel density	Univariate model				Multivariate model			
	B	β	95% CI	P value	B	β	95% CI	P value
Age, y	0.003	0.007	−0.069 to 0.075	0.934				
Sex, male/female	−0.143	−0.014	−1.778 to 1.493	0.863				
Hypertension, n (%)	−0.080	−0.006	−2.375 to 2.215	0.945				
Diabetics, n (%)	0.035	0.001	−6.908 to 6.979	0.992				
Migraine, n (%)	−0.282	−0.020	−2.577 to 2.013	0.808				
Refractive surgery, n (%)	1.773	0.094	−1.269 to 4.814	0.251				
CCT, $\mathrm{\mu m}$	0.012	0.090	−0.011 to 0.035	0.306				
Disc hemorrhage, n (%)	−1.159	−0.082	−3.446 to 1.129	0.318				
Axial length, mm	0.299	0.109	−0.174 to 0.773	0.214				
IOP mean, mmHg	−0.340	−0.172	−0.840 to 0.159	0.178				
IOP fluctuation, mmHg	−0.148	−0.069	−0.492 to 0.197	0.398				
**Visual field**								
MD, dB	−**0.158**	−**0.160**	−**0.317 to 0.002**	**0.053**	−0.084	−0.085	−0.325 to 0.157	0.491
PSD, dB	**0.184**	**0.160**	−**0.002 to 0.371**	**0.053**	0.128	0.111	−0.154 to 0.410	0.371
**Heart rate variability**								
SDNN	−0.010	−0.037	−0.053 to 0.033	0.651				
LH Norm	4.537E	0.041	0.000 to 0.000	0.619				
HF Norm	0.029	0.135	−0.006 to 0.064	0.100				
LF/HF ratio	−**0.243**	−**0.243**	−**1.219 to **−**0.261**	**0.003**	−**0.749**	−**0.249**	−**1.193 to **−**0.249**	**0.002**

B = non-standardized coefficient; β = standardized coefficient; CI = confidence interval; CCT = central corneal thickness; IOP = intraocular pressure; MD = mean deviation; PSD = pattern standard deviation;

Statistically significant values appear in boldface.

## Discussion

We have demonstrated the association between impaired deep parapapillary vessel density and autonomic dysfunction in NTG. This study showed that autonomic dysfunction has a relationship with ocular blood flow, other than that with systemic blood circulation, which has been reported in other studies. Lower parapapillary choroidal VD is a risk factor for progression of glaucoma^22–24^. Our findings agree with reports proposing that autonomic dysfunction contributes to the pathophysiology of glaucoma in terms of BP or blood flow instability, and this study is, to our knowledge, the first study to directly view VD changes using OCT-A.

In our study, the poor MD of VF correlated with IOP and parapapillary vessel density, respectively. Howerver, impaired parapapillary choroidal VD was associated with autonomic dysfunction, independent of IOP. This result may support non-mechanical theory (IOP-independent mechanism) of glaucoma pathogenesis.

Impaired blood flow is considered to be an important risk factor for glaucoma^1,2^. According to the Collaborative Normal-Tension Glaucoma Study (CNTGS) and the Early Manifest Glaucoma Trial (EMGT), migraine and disc hemorrhage contribute to the progression of VF loss^29,30^. Migraine was characterized as a vasospastic disorder and represented systemic blood flow disorders^7^. Disc hemorrhage is a local indicator of ocular blood flow disturbance^6^. With the advent of OCT-A, this concept could be directly visualized in terms of VD. Glaucomatous eyes with disc hemorrhage tended to show microvascular dropout or VD reduction on OCT-A^22,31^. According to other OCT-A studies, systemic conditions such as nocturnal BP dip, higher systemic BP or low arterial BP were associated with reduced ocular VD^27,28^. These all indicate disturbances or instability of systemic or local blood flow, as capsured by OCT-A, and this tool could be promising in terms of evaluating the vascular status of the retina or ONH in glaucoma.

Blood circulation is basically regulated by the ANS^32^. Autonomic dysfunction may contribute to unstable or fluctuating blood pressure^32^. Ocular blood flow is controlled by direct autonomic and indirect autoregulatory system. Vasculature of the optic nerve, choroid, ciliary body, and iris were controlled by direct autonomic system. Retinal blood flow was controlled by indirect autoregulatory system^33^. Abnormality of the ANS cause disturbance of the blood supply to the ONH and choroid^34^. Given that the choroid contributes to the prelaminar blood supply of the ONH, decreased choroidal blood flow may promote progression of glaucoma^35,36^. Choroidal blood flow is innervated by the parasympathetic and sympathetic nervous systems^36^. These vessel are innervated by postganglionic nerve fibers from the pterygopalatine (parasympathetic) and superior cervical (sympathetic) ganglia^33,36^.Unlike the choroidal circulation, there is no neuronal innervation in the retinal vessel^37^. The physiology and anatomy of the retinal circulation are similar to the brain circulation^37,38^.The presence of endothelial tight junctions results in the blood-retinal barrier, resembling the blood–brain barrier^37,38^.Histological studies have demonstrated a abundant autonomic innervation for the choroid, but these nerves do not go further into the retina^39^. Hence, retinal blood flow is mainly controlled through autoregulation by myogenic and local metabolic mechanisms^38,39^. The mediators of these mechanisms include oxygen, adenosine, nitric oxide, angiotensin-II, carbon dioxide, and endothelin-1^37^. Both superficial and deep macular vessels are supplied by retinal vessels. Radial peripapillary capillaries (RPCs) are mainly supplied by retinal blood flow. Only the deep parapapillary blood flow is controlled by choroidal vessels, which are innervated by the ANS. Hence, autonomic function should show an association with the status of the choroidal vessels and our results show that HRV, an indicator of ANS function, was only correlated with parapapillary choroidal VD.

The vascular plexus of the superficial macular layer is located mainly within retinal nerve fiber layer (RNFL), retinal ganglion cell (RGC) layer and inner plexiform layer (IPL). Measuring macular perfusion has the potential for detecting reduced metabolic rate and dysfunction of the RGCs. The superficial parapapillary layer contains RPCs, which compose a unique capillary plexus within the RNFL, and constitute a critical blood source to satisfy the nutritional demands of RGC axons^40^. Because glaucoma damages RGCs and the RNFL, superficial macular and parapapillary perfusion could theoretically be used to assess disease severity^41,42^. Previous studies reported that superficial VD was correlated with MD of VF and the macular RGC layer and IPL^41,42^. Our results also showed that the MD of VFs is correlated with macular and parapapillary superficial VD. Changes of superficial VD could be the result of RNFL loss and, thus, ANS did not show correlation with superficial VD parameters.

ANS function can be measured by a variety of methods. Among the many relevant procedures, HRV analysis has rapidly expanded in use in the investigation of both normal physiology and pathologic conditions. HRV has also been used to evaluate ANS function in glaucoma patients. A 24-h HRV analysis showed that there was increased sympathetic activity of the ANS in patients with NTG, and that the extent of the autonomic disorder correlated with the severity of glaucoma^43,44^. Short term HRV analysis showed that patients with NTG had an autonomic imbalance that was related to serum levels of endothelin-1^15,19,45,46^. Because the imbalance between the sympathetic and parasympathetic autonomic systems (i.e., the LF/HF ratio in our study) had a significant association with deep parapapillary VD, we can state that autonomic dysfunction influences choroidal blood flow around the ONH, which may contribute to the progression of glaucoma in patients with autonomic imbalance.

**Figure 2:**
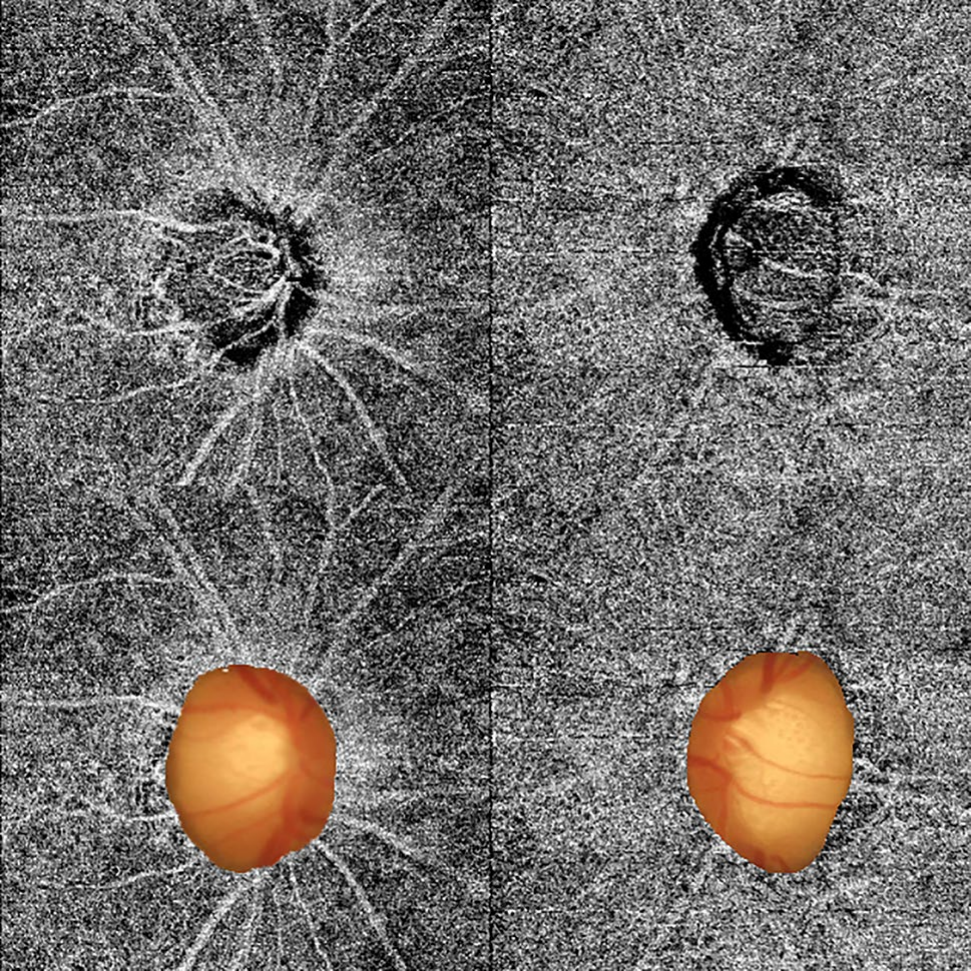
Two representative cases with contrasting features. Left image is low parapapillary choroidal vessel density with high LF/HF ratio (5.41). Right image is high parapapillary choroidal vessel density with low LF/HF ratio (2.18). The vessel density was measured in whole area except for the optic disc region.

Representative cases are shown in Fig. 2. The picture on the left is the eye of a patient with a high LF/HF ratio (5.41). The VD was measured in the area except for the optic disc region. The optic disc area can be seen in the figure below. The picture on the right is the eye of a patient with a low LF/HF ratio (2.18). The calculated choroidal VD was lower in the left one than in the right one. (47.92% vs 56.61%, respectively).

In our study, the poorer MD or PSD of VF was correlated with the lower parapapillary vessel density, which is consistent with previous reports. Howerer, multivariate analysis did not show statistical significance. This is because this study is a cross-sectional study, not a longitudinal study. The impaired blood flow increases the risk of glaucoma progression, but does not necessarily mean that the current glaucoma damange is severe. Another possibility is that the glaucoma damage in this study was an early stage.

There are limitations in this study. First, this study has the intrinsic limitation of a cross-sectional design described above. Second, the VD obtained by OCTA may not perfectly match to blood flow due to their floor effect. Third, we could not exclude the patients with DM that could affect the HRV. However, only two diabetic patients were included in this study. Forth, we could not totally exclude the patients with cataract or multifocal intraocular lens insertion that could affect the quality of OCTA image.

In summary, impaired parapapillary choroidal blood flow was associated with autonomic dysfunction. This study supports the association between glaucomatous damage and autonomic dysfunction, and its possible role in glaucoma progression.

## Materials and methods

### Study subjects

This study included 152 normal tension glaucoma patients (152 eyes) who visited the Seoul St. Mary's Hospital between January 1, 2020 and December 31, 2020. Institutional Review Board (IRB)/Ethics Committee approval was obtained from the Seoul St. Mary’s hospital’s IRB, which waived the requirement for written informed consent because of the retrospective nature of the study. All relevant principles of the Declaration of Helsinki were followed. All patients had a detailed ophthalmic examination, including slit lamp examination, measurements of IOP (Goldmann applanation tonometry), central corneal thickness (UD-800; Tomey Corporation, Japan), axial length (IOL Master; Carl Zeiss Meditec, Dublin, CA), red-free fundus photography (Canon; Tokyo, Japan), mean RNFL thickness (Cirrus OCT; Carl Zeiss Meditec) and a VF test (Humphrey field analyzer using the Swedish Interactive Threshold Standard 24–2 algorithm; Carl Zeiss Meditec). Histories of hypertension, migraine, diabetes and optic disc hemorrhage were investigated through medical records. Patients taking drugs known to affect the ANS, such as beta-blockers and digoxin, were excluded. Glaucomatous VF defects were defined by the following findings: 1) a cluster of ≥ 3 adjacent points in pattern deviation plots with a probability of < 5% of the normal population, with one of these points having a probability of < 1%, or a cluster of ≥ 2 adjacent points in pattern deviation plots with a probability of < 1%; 2) glaucoma hemifield test results outside the normal limits and PSD < 5%.

The mean IOP was the average of all measurements taken for 3 years preceding th last visit. IOP fluctuation was calculated as the difference between the maximum and minimum IOP values obtained during the follow-up period.

### HRV assessment

The participants were asked to avoid activities such as running, jumping, ect at least 2 h before the test. The testing was done over 5 min in very-controlled conditions. The echocardiography was monitored by an experienced technician for 5 min, and the echocardiography signals were transferred to a Medicore Heart Rate Analyzer, Model SA-3000P (Medicore, Seoul, Korea).

The short-term HRV allow to obtain and accurate and meaningful data under more controlled conditions reducing confounder factors, compared to the long-term HRV^47–49^. The reproducibility of short-term HRV parameters has been demonstrated in previous human cohorts studies^48^.

The standard deviation of the NN-interval (SDNN) index is obtained by measuring the normal R-R interval of QRS complex in the electrocardiogram. Both the sympathetic and parasympathetic nervous systems contribute to the SDNN^13^. A reduction in SDNN is correlated with left ventricular dysfunction, indicating a high tone of heart sympathetic activity and has been found to be predictive of increased risk of sudden cardiac death^13^.

The power in the HF is the parameter of the spectral power of heart rate respiratory undulations reflecting the activity level of the respiratory center^50,51^. The HF band reflects fast changes in beat-to-beat variability, which are due to the parasympathetic activity^50,51^ The power in the LF modulated by both the sympathetic and parasympathetic nervous system, but LF band is considered to be a sympathetic activity^50,51^. It reflects the activity of the baroreflex mechanism that ensures BP regulation^50,51^. “LF/HF” is the ratio of LF power to HF power and reflects the sympathetic-vagal balance. A higher ratio indicates increased sympathetic activity or reduced parasympathetic activity^52^. Both the SDNN and LF/HF ratio are affected by the sympathetic and parasympathetic nervous systems, but the LF/HF ratio more accurately represents the magnitude and direction of autonomic imbalance^52^.

### OCT angiography

The macula and ONH were scanned using a swept-source OCT-A device. Scans were taken with 4.5-cm × 4.5-cm cubes. Identification of the microvasculature was based on automated layer segmentation, performed by the built-in OCT software. Each cube consisted of 320 clusters of four repeated B-scans centered on the optic disc or macular region.

The superficial macular microvasculature reaches from 2.6 μm below the internal limiting membrane (ILM) to 15.6 μm below the junction of the IPL and inner nuclear layer (INL) (IPL/INL). The deep macular microvasculature reaches from 15.6 μm below IPL/INL to 70.2 μm below IPL/INL. The vascular plexus of the superficial macular layer is located mainly within the RNFL, RGC layer and IPL. The vascular plexus of the deep macular layer is located within the INL^53^.

The superficial parapapillary microvasculature reaches from the ILM to the RNFL. The deep parapapillary microvasculature reaches from 130 μm below the ILM to 390 μm below the basement membrane.

Measurement of VD from OCT-A images was performed as described in previous studies^54–56^ The binarized image was converted to a red–green–blue color model and then split into three channels (red, green and blue). The red channel was chosen as the reference. After using the “adjust threshold” tool, which automatically sets the lower- and upper-threshold values, images were segmented into the area of interest and the background. The white pixels represented “vessel” and black pixels represented “background”. The VD was calculated as the percentage of the white-pixels area, divided by the total image area.

### Statistical analysis

We Sample size calculations were performed using a statistical power analysis program (G*Power 3.1 software). At the significance level of 5% and minimum power of 95% and considering a minimum value of 0.3 for correlation, 138 individuals are required in the sample. Data are presented as means and SDs. Pearson correlation analysis was used to evaluate the relationships between VD and the autonomic dysfunction parameters, such as SDNN, HF, LF and the HF/LF ratio. The linear and logarithmic R^2^ values were calculated from scatter plots of VD and the LF/HF ratio of HRV. Univariate and multivariate logistic regression analyses were used to identify factors associated with parapapillary choroidal VD. Values of P < 0.05 in the univariate model were included in the multivariate model. Values of P < 0.1 indicated statistical significance. All statistical analyses were performed using SPSS for Windows (v. 24.0; IBM Corporation, Armonk, NY).
